# Exploring potential imaging biomarkers for ischemic retinal vein occlusion: insights from fundus autofluorescence and optical coherence tomography

**DOI:** 10.3389/fmed.2025.1726962

**Published:** 2025-12-31

**Authors:** Zixun Wang, Jun Zhou, Xiaoxue Hu, Jingjie Ding, Xiaoling Zhang, Ruihua Wei, Xiuhong Qin, Zhiqing Li

**Affiliations:** 1 Tianjin Key Laboratory of Retinal Functions and Diseases, Tianjin Branch of National Clinical Research Center for Ocular Disease, Eye Institute and School of Optometry, Tianjin Medical University Eye Hospital, Tianjin, China; 2 The First Affiliated Hospital of Dalian Medical University, Dalian, Liaoning, China; 3 Wuhan Children’s Hospital (Wuhan Maternal and Child Healthcare Hospital), Tongji Medical College, Huazhong University of Science and Technology, Wuhan, Hubei, China; 4 Handan Eye Hospital (The Third Hospital of Handan), Hebei, China

**Keywords:** disorganization of retinal inner layers, fundus autofluorescence, optical coherence tomography, prominent middle-limiting membrane, retinal vein occlusion

## Abstract

**Purpose:**

This study aimed to investigate the characteristics of macular edema observed by fundus autofluorescence (FAF) and to analyze noninvasive indicators of ischemia in retinal vein occlusion (RVO) through FAF or optical coherence tomography (OCT). We specifically report a newly recognized FAF–OCT mismatch in ischemic RVO, in which CME is not detectable on FAF.

**Methods:**

This study included 74 participants. 49 eyes of 49 patients with RVO and 25 healthy control eyes were included in the study. All patients underwent a complete ophthalmologic examination and were evaluated with FAF, fundus fluorescein angiography (FFA), and OCT. The optical intensities of the inner and outer retinal layers were measured using ImageJ software.

**Results:**

Of 49 eyes in RVO patients, FAF imaging showed typical Cystoid macular edema (CME) with hyperautofluorescence in non-ischemic RVO patients (69.57%) and in ischemic RVO patients (26.92%, *p* = 0.029, *p* < 0.05). Disorganization of retinal inner layers (DRIL) was observed in 13 eyes in the non-ischemic RVO group and 23 eyes in the ischemic RVO group. (*χ ^2^*= 6.387, *p* = 0.012, *p* < 0.05) The prominent middle-limiting membrane (p-MLM) sign was found in 5 eyes in the non-ischemic RVO group and 15 eyes in the ischemic RVO group. (*χ ^2^*= 6.530, *p* = 0.011, *p* < 0.05) The mean optical intensity of the retinal pigment epithelium (RPE) layer, ellipsoid, and interdigitation zones in the ischemic RVO group was 109.78 ± 19.327, which was significantly lower compared to the non-ischemic RVO group (172.31 ± 30.527, *p* = 0.001, *p* < 0.01).

**Conclusion:**

CME in patients with ischemic RVO is less easily detectable on FAF compared to patients with non-ischemic RVO. This lack of correspondence may provide insight into the type of RVO. We conclude that the presence of DRIL, a p-MLM sign, impairment of the integrity of the external limiting membrane (ELM), and decreased optical intensity or integrity of the ellipsoid zone and RPE can serve as effective indicators of ischemia in RVO patients.

## Introduction

Retinal vein occlusion (RVO) is a common retinal vascular disease that leads to visual impairment (1). RVO is characterized by obstruction of the retinal venous system and often occurs in older people (1, 2). While the pathogenesis of RVO remains incompletely understood, it is believed to result from thrombosis of the retinal veins (3). The risks of stroke, myocardial infarction, deep vein thrombosis, pulmonary embolism, and death following RVO are also increasingly recognized (4). In the elderly, risk factors may include hypertension, diabetes, arteriosclerosis, cardiovascular and cerebrovascular diseases, or open-angle glaucoma (5, 6). In the young, RVO was associated with hematological changes and local or systemic inflammation (6).

Retinal vein occlusion (RVO) has been classified into central RVO (CRVO), branch RVO (BRVO), and hemi RVO based on the different locations of the venous obstruction (7). Fundus fluorescence angiography (FFA) has been used for classifying cases of ischemic and non-ischemic RVO based on the retinal non-perfusion areas (7). Different types of RVO have distinct therapeutic schedules and visual prognoses. Traditional FFA, as an invasive examination, can generally reveal macular edema by fluorescein accumulation on the Henle fibers, which RVO induces. Optical coherence tomography (OCT), a non-invasive imaging technique, has also been clinically applied to observe the retinal and choroidal microstructure. In recent years, an increasing number of studies have focused on OCT morphological parameters, such as hyperreflective foci (HF), disorganization of retinal inner layers (DRIL), subretinal fluid (SRF), disorganization of the external limiting membrane (ELM), and the ellipsoid zone (EZ), to predict the visual prognosis of RVO patients (7, 8). Macular edema, the leading cause of vision loss in RVO, can also be clearly visualized on OCT. Multimodal imaging recognition technology for RVO has also achieved significant breakthroughs (9, 10). However, for the diagnosis of ischemic RVO, fluorescein angiography (FFA) remains the gold standard.

Fundus autofluorescence (FAF) is a type of non-invasive fundus examination that can show the content and distribution of lipofuscin in the retinal pigment epithelium (RPE) and reflects the function and metabolism of RPE cells (11). FAF is often used to assist in diagnosing age-related macular degeneration (AMD), uveitis, or retinitis pigmentosa (12). However, few studies on cystoid macular edema (CME) have used FAF due to interference from the macular pigment (13). We discovered that whereas CME on FAF images is often not particularly visible, it is highly evident in clinical practice. Elaborating on the features and uses of CME could be an intriguing perspective from the FAF’s viewpoint while monitoring CME in RVO patients.

In this study, we aimed to investigate the image features of FAF, FFA, and OCT in patients with different types of RVO and to analyze the possible pathologies.

## Materials and methods

### Ethical approval and study design

This study was approved by the ethics committee of the First Affiliated Hospital of Dalian Medical University and Tianjin Medical University Eye Hospital [No: PJ-KS-KY-2023-288; KY-202467]. All study procedures adhered to the tenets of the Declaration of Helsinki. All procedures involving comprehensive physical examinations and ophthalmic assessments were performed with participants’ informed consent.

Consecutive patients with RVO at the Department of Ophthalmology, the First Affiliated Hospital of Dalian Medical University, and Tianjin Medical University Eye Hospital, China, between December 2021 and October 2024 were included. A total of 25 age-matched healthy control eyes of patients from our institution were included in this study. Healthy control participants had no systemic diseases such as diabetes or hypertension.

The diagnosis of RVO was based on the following criteria: (1) RVO had to be detected through fundus examination and FFA; and (2) the patients with RVO, including CRVO, BRVO, or HRVO, had to be treatment naïve. The exclusion criteria were as follows: (1) macular edema secondary to other retinal diseases, such as diabetic retinopathy, AMD, uveitis, exudative retinal detachment, or choroid neoplasms; (2) other retinal diseases, such as epimacular membrane, central serous chorioretinopathy, or polypoidal choroidal vasculopathy; and (3) history of retinal or cataract surgeries.

Basic information, including age, gender, time of onset, and history of systemic diseases, was recorded for all patients with RVO. Patients were divided into two groups based on FFA: those with non-ischemic RVO and those with ischemic RVO. Ischemic RVO is defined as a retinal capillary non-perfusion area on FFA imaging that is ≥10 disc areas. This constitutes the gold standard for diagnosis (14). Normal controls were selected from healthy individuals based on age distribution. All patients and control subjects underwent complete ophthalmologic examinations, which included tests for best-corrected visual acuity (BCVA), autorefraction, dominant eye examination, intraocular pressure, slit lamp examination, fundoscopy, and FFA examination.

### Dominant eye test

The card-hole method was used to test the dominant eye. The patient was asked to hold a card with a 3 cm diameter hole, straighten their arms, and gaze through the hole at a distant visual target with both eyes. Then, the patient needed to close each eye alternately; if the visual target disappeared after closing one eye, that eye was the dominant eye.

### FAF, OCT examination, and measurement of optical intensity

Clear FAF images were obtained by superimposing ART images with confocal scanning laser angiography (Heidelberg Engineering, Heidelberg, Germany). Then, FFA was performed, during which 5 ml of sodium fluorescein was injected intravenously into the patient’s ulnar vein. Cross-sectional images of the macular and lesion areas were obtained using Spectral OCT (Heidelberg Engineering, Heidelberg, Germany). A dense volume scan with 30 average B-scans per image was also performed on the lesion areas.

All OCT/FAF biomarkers were defined using standardized operational criteria. DRIL was defined as loss of identifiable boundaries between the IPL, INL, and OPL over ≥250 μm in the central 1-mm zone. The p-MLM sign was defined as a continuous hyper-reflective line at the outer INL border independent of overlying cysts. The HF is defined as ≤30 μm hyperreflective dots without backshadowing. The ELM/EZ/IZ disruption was defined as focal loss ≥100 μm or diffuse attenuation of reflectivity. The FAF–OCT mismatch was defined as the presence of OCT-confirmed cystoid macular edema without corresponding hyperautofluorescence on FAF in the same region. Two masked graders independently assessed all biomarkers. Inter-grader agreement was good (ICC range: 0.89–0.92).

The central macular thickness (CMT) was measured in the RVO and control eyes. The CMT was defined as the vertical distance from the nerve fiber layer to the RPE layer. The inner retinal layer (IRL) contains the ganglion cell layer, inner plexiform layer, inner nuclear layer, and outer plexiform layer. The outer retinal layer (ORL) contains the ELM, ellipsoid layer, and RPE layer. All OCT and FAF-related features were identified by two experienced ophthalmologists, who performed this task without knowledge of the RVO type. The optical intensity of these parameters was measured using ImageJ software, as previously described (10). Optical intensity was quantified using ImageJ (NIH, USA). For all OCT images, the following standardized procedure was applied: (1) ROI selection and anatomical standardization: all measurements were taken on the horizontal foveal scan; ROIs were manually drawn at three fixed anatomical locations to ensure reproducibility, including foveal center, 500 μm nasal to the fovea, and 500 μm temporal to the fovea; for each area, rectangular ROIs (width 200 μm, height adjusted to layer thickness) were placed on the outer retina, including the ONL, OPL, and photoreceptor layers. (2) Image normalization: To control signal strength and inter-image brightness variation, all OCT images were normalized using the vitreous cavity as the reference (grey value = 0) and the RPE band as the internal high-reflectivity reference. The following normalization formula was applied: Normalized intensity = (Measured gray value -Vitreous gray value) / (RPE gray value – Vitreous gray value). (3) Measurement parameters: The mean gray value within each ROI was automatically extracted. For each eye, the average of the three locations was used for statistical analyses. (4) Inter-observer reliability: Two masked graders independently measured all images. Inter-observer reliability was assessed using intraclass correlation coefficients (ICCs), which showed excellent agreement (ICC = 0.92 for outer retinal intensity and ICC = 0.89 for inner nuclear layer intensity). Choroidal thickness was measured using the EDI mode on OCT. Subfoveal Choroidal Thickness (SFCT) was defined as the vertical distance between the hyperreflective lines of the RPE to the choroid/sclera border at the central fovea. The SFCT was measured twice by two observers using the OCT system software. The mean of the two measurements of each patient and control subject was used for the analysis.

### Statistical analysis

SPSS 23.0 software was used for statistical analysis. The results of the basic information and FAF/OCT examinations were compared using a Chi-square test (χ2). Normality was tested using the Shapiro–Wilk test, and homogeneity of variances was evaluated using Levene’s test before conducting ANOVA. Only variables that satisfied both assumptions were analyzed using parametric tests. All *P*-values < 0.05 were considered statistically significant.

## Results

### Basic information

Forty-nine eyes from 49 patients with RVO were included in the study. Of our 49 patients, 23 (46.94%) were male, and 26 (53.06%) were female. The ages of the patients with RVO ranged from 24 to 73 years (mean age 54.4 years). The cause of the disease ranged from 1 day to 3 years. All these patients presented with unilateral RVO, with 18 patients having CRVO and 31 patients having BRVO. Of the 49 eyes, 23 (46.94%) presented with non-ischemic RVO, and 26 (53.06%) presented with ischemic RVO. In the non-ischemic RVO group, 12 patients presented with CRVO, and 11 patients with BRVO. In the ischemic RVO group, six patients presented with CRVO and 20 with BRVO. We selected 25 age-matched eyes as the control group, with a mean age of 53.8 years (range 28–73 years). Of these 49 patients, two had diabetes, 20 had hypertension, eighteen had a history of alcohol intake, 15 had a history of smoking, 17 had hyperlipidemia, one had psoriasis, 4 had coronary disease, and twelve had no history of diseases. There was no statistically significant difference in age, gender, and laterality among the three groups. The demographic information for the non-ischemic RVO and ischemic RVO groups is summarized in Table 1.

**TABLE 1:** The basic information of non-ischemic and ischemic RVO groups (eyes).

Basic information	Ischemic RVO	Proportion	Non-ischemic RVO	Proportion	*χ^2^*	*P-*value
Total number	26	53.06%	23	46.94%		
Gender					0.208	0.648
Male	13	50%	10	42.48%		
Female	13	50%	13	56.52%		
Laterality					1.417	0.234
Right eye	11	42.31%	6	26.09%		
Left eye	15	57.69%	17	72.91%		
RVO types					3.299	0.069
BRVO	20	76.92%	12	52.17%		
CRVO	6	23.08%	11	47.83%		
Smoke	8	30.77%	7	30.43%	0.001	0.975
Drinking	7	26.92	11	43.83%	2.294	0.130
Hypertension	10	38.46%	10	43.48%	0.127	0.722
Diabetes	1	3.85%	1	4.35%	0.008	0.929
Coronary disease	3	11.54%	1	4.35%	0.842	0.359
Hyperlipidaemia	9	34.62%	8	34.78%	<0.001	1.000
Dominant eye	8	30.77%	6	26.09%	0.131	0.717

### Dominant eye in the RVO

Among these 49 patients, 14 had the condition affecting their dominant eye. The proportion of RVO that occurred in the dominant eye was about 28.57%. Six (26.09%) patients in the non-ischemic RVO group had the affected eye as their dominant eye, and eight (30.77%) patients in the ischemic RVO group had the same condition. However, there was no statistically significant difference among the RVO groups (*p* > 0.05).

### Manifestations of FAF in RVO patients

**FIGURE 1:**
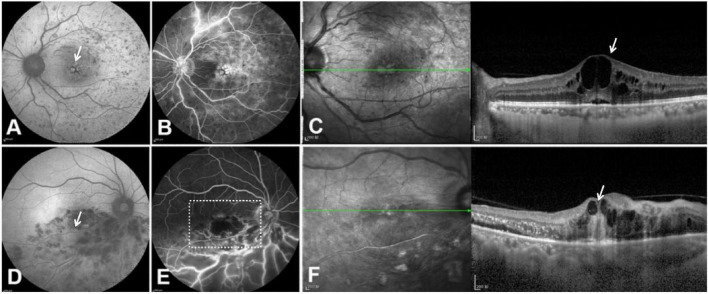
FAF, FFA, and OCT images of non-ischemic RVO **(A–C)** and ischemic RVO **(D–F)**. **(A)** In non-ischemic RVO, FAF showed hyperautofluorescence, indicative of macular edema (white arrow). **(B)** FFA revealed late-stage fluorescence accumulation in the macula. **(C)** OCT showed a cystic cavity at the inner retina in the macula of the left eye (white arrow). **(D)** In ischemic RVO, FAF did not reveal macular edema (white arrow). **(E)** FFA showed non-perfusion areas in the macula without fluorescence leakage (white box). **(F)** OCT showed inner retinal macular edema in the right eye of the same patient (white arrow). FAF, fundus autofluorescence; FFA, fundus fluorescein angiography; OCT, optical coherence tomography; RVO, retinal vein occlusion.

In the non-ischemic RVO group (23 eyes), FAF imaging showed typical macular cystoid edema with hyperautofluorescence (Figure 1A) in 16 patients (69.57%). All these patients exhibited typical macular cystoid edema on late-stage FFA (Figure 1B) and cystic cavities within the inner retina in the macular region on OCT (Figure 1C). There was no spontaneous fluorescence observed in the macular area, among which 4 cases of CRVO had no macular edema, and 3 cases with BRVO involved no macular region. In the ischemic RVO group (26 eyes), FAF imaging revealed macular edema with hyperautofluorescence in only seven patients (26.92%, *p* = 0.029, *p* < 0.05). Among the remaining 19 patients, hyperautofluorescence in the macula was observed on FAF (Figure 1D). Non-perfusion areas were observed in the macula in 11 eyes (Figure 1E), despite OCT showing inner retinal macular edema (Figure 1F). The macular region was not involved in five eyes, and a thick macular hemorrhage was found in 3 eyes.

**FIGURE 2:**
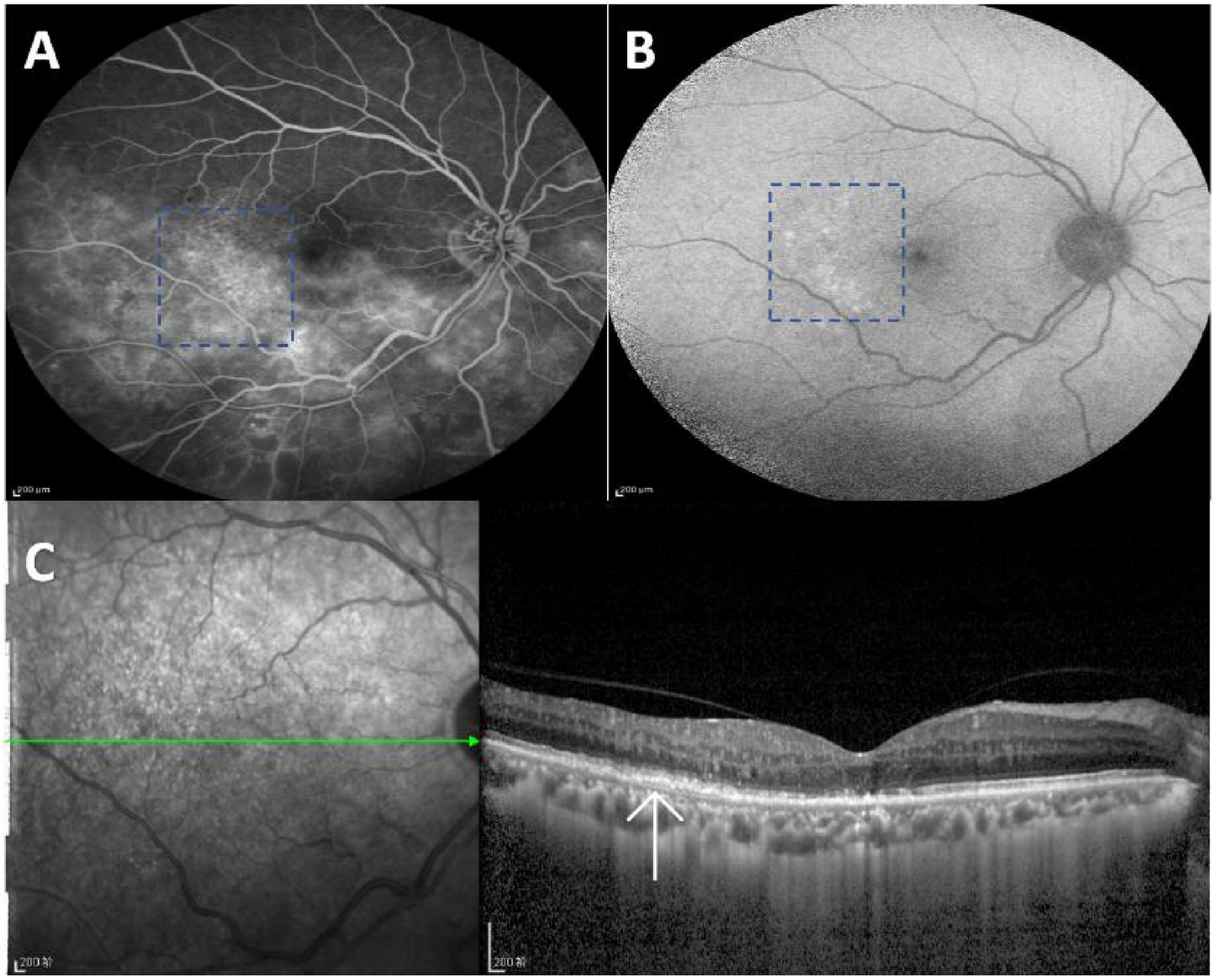
RPE changes were observed in the right eye of a single patient with ischemic RVO. **(A)** FFA revealed a window defect in the temporal macular area(synchronous with choroidal fluorescence). **(B)** FAF showed scattered dots of hyperautofluorescence in the temporal macular area. **(C)** OCT showed RPE changes in the temporal macula (indicated by the white arrow). FAF, fundus autofluorescence; FFA, fundus fluorescein angiography; OCT, optical coherence tomography; RVO, retinal vein occlusion; RPE, retinal pigment epithelium; BRVO, branch retinal vein occlusion; CRVO, central retinal vein occlusion.

In all 49 eyes, hypo autofluorescence was observed in the non-perfusion areas, and retinal hemorrhage appeared as hypo autofluorescence in FAF. Only one patient, with a 2-year onset, exhibited scattered dot hyperautofluorescence in the temporal macular area (Figure 2A), which was accompanied by RPE layer abnormalities on FFA (Figure 2B) and OCT (Figure 2C).

Fundus autofluorescence (FAF) imaging can also reveal papilledema in different types of RVO. In the non-ischemic RVO group, 8 patients (8 eyes, 34.78%) had papilledema, and FAF imaging showed an unclear, low-autofluorescence boundary of the optic disk, which was associated with leakage of fluorescence on FFA. In the ischemic RVO group, only 4 patients (4 eyes, 15.38%) had papilledema, fewer than in the non-ischemic RVO group.

### Manifestations of different-type RVO patients in OCT examination

The OCT examination revealed intraretinal cysts and subretinal fluid in patients with different types of RVO. In the non-ischemic RVO group, intraretinal cysts were found in 18 eyes (78.26%) and subretinal fluid in fifteen eyes (65.22%). In the ischemic RVO group, 23 eyes (88.46%) presented with intraretinal cysts, and 16 eyes (61.54%) presented with subretinal fluid (*p* > 0.05). The ratio of intraretinal cysts was high in both groups, and the two groups had similar proportions of intraretinal cysts and subretinal fluid. The OCT also revealed multiple hyperreflective bright spots in the inner retina in most eyes, which were more common in the ischemic RVO group than in the non-ischemic RVO group. In the non-ischemic RVO group, multiple hyperreflective bright spots were found in 19 eyes (82.61%), and there were 26 eyes (100%) with the same condition in the ischemic RVO group.

An important OCT parameter of DRIL was found in the RVO patients. We found that 13 eyes (56.52%) in the non-ischemic RVO group and 23 eyes (88.46%) in the ischemic RVO group had DRIL. The DRIL ratio was significantly higher in the ischemic RVO group than in the non-ischemic RVO group. (*χ ^2^*= 6.387, *p* = 0.012, *p* < 0.05) The degree of the DRIL was more serious in the ischemic RVO group. Parts of the patients’ eyes had outer limiting membrane (ELM) integrity, ellipsoid, and interdigitation zones damaged in the lesion areas. In our 49 patients, thirteen eyes had ELM integrity damaged, among which three were in the non-ischemic RVO group, and ten were in the ischemic RVO group. (*χ ^2^*= 4.045, *p* = 0.044, *p* < 0.05) Meanwhile, 18 eyes presented with the disappearance or disorder of the ellipsoid and interdigitation zones, among which seven eyes were found in the non-ischemic RVO group, and eleven eyes were in the ischemic RVO group. RPE changes were rarely observed in RVO patients, and only 3 eyes were found to have RPE abnormalities in the ischemic RVO group.

The OCT revealed a prominent middle-limiting membrane (p-MLM) in both groups. Five eyes (21.74%) had p-MLM in the non-ischemic RVO group, and fifteen eyes (57.69%) had the same condition in the ischemic RVO group. The ratio of the presence of p-MLM was significantly higher in the ischemic RVO group than in the non-ischemic RVO group. (*χ^2^*= 6.530, *p* = 0.011, *p* < 0.05) The morphological biomarkers in the two RVO groups are summarized in Table 2.

**TABLE 2:** OCT and FAF features in ischemic and non-ischemic RVO.

OCT/FAF features	Ischemic RVO (number of eyes)	Non-ischemic RVO (number of eyes)	*χ^2^*	*P*
Intraretinal cyst	23	18	0.930	0.335
Subretinal fluid	16	15	0.071	0.790
DRIL	23	13	6.387	0.012
Hyperreflective bright spots	26	19	4.924	0.026
ELM integrity impairment	10	3	4.045	0.044
Ellipsoid and interdigitation zones changes	11	7	0.740	0.390
RPE changes	3	0	2.827	0.093
PVD	9	14	3.377	0.066
p-MLM sign	15	5	6.530	0.011
CME hyper fluorescence on FAF	16	7	4.740	0.029

OCT, optical coherence tomography; RVO, retinal vein occlusion; DRIL, disorganization of retinal inner layers; ELM, outer limiting membrane; RPE, retinal pigment epithelium; PVD, posterior vitreous detachment; p-MLM, prominent middle-limiting membrane.

The mean CMT in the non-ischemic RVO group was 480.57 ± 235.514 μm, which was significantly higher than the mean CMT of 224.53 ± 16.977 μm in the control group (*p* = 0.001, *p* < 0.01). The mean CMT in the ischemic RVO group was 403.69 ± 179.329 μm, which was also significantly higher than the mean CMT of the control group (*p* = 0.016, *p* < 0.01). There was no significant difference in CMT between the non-ischemic RVO group and the ischemic RVO group (*p* = 0.667, *p* > 0.01).

The mean SFCTs in the non-ischemic RVO group, ischemic RVO group, and control group were 259.04 ± 61.151 μm, 250.97 ± 67.682 μm, and 265.80 ± 52.705 μm, respectively. There was no significant difference in the SFCT among these three groups (*p* = 0.794, *p* > 0.01).

### Measurement of optical intensity of different layers of the retina on OCT

All ANOVA variables, including CMT and outer retinal optical intensity, passed the normality test (Shapiro–Wilk *p* > 0.05) and homogeneity of variance test (Levene’s *p* > 0.05). In the ischemic RVO group, the mean optical intensity of the IRL was 160.51 ± 21.916, which was significantly higher compared to the normal control group (130.7 ± 26.826, *p* = 0.001, *p* < 0.01). The mean optical intensity of the RPE layer, ellipsoid, and interdigitation zones in the ischemic RVO group was 109.78 ± 19.327, which was significantly lower compared to the normal control group (184.70 ± 18.462, *p* = 0.002, *p* < 0.01).

In the non-ischemic RVO group, the mean optical intensity of the IRL was 155.34 ± 14.806, which was significantly higher compared to the normal control group (130.7 ± 26.826, *p* = 0.013, *p* < 0.05). There was also no significant difference in the optical intensity of the RPE layer, ellipsoid, and interdigitation zones between the non-ischemic RVO group (172.31 ± 30.527) and the control group (184.70 ± 18.462, *p* = 0.471, *p* > 0.05).

Furthermore, there was no significant difference in the optical intensity of the IRL between the ischemic RVO group (160.51 ± 21.916) and the non-ischemic RVO group (155.34 ± 14.806, *p* = 0.895, *p* > 0.05). The mean optical intensity of the RPE layer, ellipsoid, and interdigitation zones in the ischemic RVO group was 109.78 ± 19.327, which was significantly lower compared to the non-ischemic RVO group (172.31 ± 30.527, *p* = 0.001, *p* < 0.01).

The measurements of the CMT, SFCT, and optical intensity of the different retinal layers in patients with RVO are summarized in Table 3.

**TABLE 3:** The data of CMT, SFCT, and optical intensity of RPE layer, ellipsoid, and interdigitation zones in ischemic and non-ischemic groups of RVO.

OCT features	Ischemic RVO	Non-ischemic RVO	Normal Control	*P*-value
CMT (μm)	403.69 ± 179.329	480.57 ± 235.514	224.53 ± 16.9777	*p* = 0.004, *p* < 0.05
SFCT (μm)	250.97 ± 67.682	259.04 ± 61.151	265.80 ± 52.705	*p* = 0.794, *p* < 0.05
Optical intensity of IRL	160.51 ± 21.916	155.34 ± 14.806	130.7 ± 26.826	*p* = 0.895, *p* > 0.05
Optical intensity of RPE layer, ellipsoid, and interdigitation zones	109.78 ± 19.327	172.31 ± 30.527	184.70 ± 18.462	*p* = 0.001, *p* < 0.05

RVO, retinal vein occlusion; CMT, central macular thickness; SFCT, subfoveal choroidal thickness; RPE, retinal pigment epithelium; IRL, inner retinal layer.

## Discussion

In our study, we identified a novel FAF finding in ischemic RVO—namely, that CME evident on OCT may not present as hyperautofluorescence on FAF due to macular ischemia and non-perfusion. This FAF–OCT mismatch has not been previously highlighted and may provide a new non-invasive clue for identifying ischemic RVO. The presence of DRIL, the p-MLM sign, impairment of ELM integrity, and decreased optical intensity or integrity of the EZ and RPE can further serve as effective indicators of ischemia in RVO patients.

Currently, no accurate standard for evaluating ischemic RVO has been established, and there is a lack of relevant research on comprehensive OCT indicators and quantitative data in patients with ischemic RVO, as well as on RVO-related indicators in FAF. Clinically, RVO can be divided into ischemic RVO and non-ischemic RVO, according to FFA examination (7). However, some patients cannot be classified due to drug allergies in the FFA examination, which can affect the treatment and prognosis of patients. Therefore, we are committed to identifying non-invasive markers to predict ischemic RVO for clinical guidance and treatment. As a non-invasive retinal examination method, OCT is widely used in clinical diagnosis and can clearly visualize retinal layer structure and the morphology of pathological changes (15). In patients with RVO, OCT can detect retinal inner layer cysts, subretinal fluid, DRIL, and macular edema (16). If the type of RVO can be determined by observing the difference in OCT parameters between patients with ischemic and non-ischemic RVO, this would provide an essential basis for guiding treatment.

In this retrospective study, we selected 49 patients with RVO from our department, including 23 with non-ischemic RVO and 26 with ischemic RVO, to investigate changes in clinical and imaging features and to determine relevant ischemic parameters. In this study, no differences were found in incidence, sex, age, or laterality between the ischemic and non-ischemic groups. We found that the proportion of CRVO was larger (52.17%), and the proportion of BRVO was relatively small in the non-ischemic RVO group. In the ischemic group, the proportion of BRVO was larger (76.92%), and all 20 cases of BRVO occurred at the intersection of the arteriovenous branches after coming out of the optic disk. This type of BRVO may be prone to retinal ischemia due to arteriovenous cross-compression within the tight vascular sheath, in which case the compression cannot be relieved—long-term hypoxia and ischemia of the retina from this compression lead to the formation of ischemic RVO. In the non-ischemic group, eleven eyes presented with BRVO, and all these cases of BRVO occurred within the vein of the optic disk. The reason these BRVOs were not prone to ischemic RVO may be that they were not completely obstructive or compressive. Therefore, we concluded that BRVO caused by arteriovenous compression is more prone to ischemic changes. Accordingly, the papilledema rate in non-ischemic RVO was 34.78%, higher than in ischemic RVO (15.38%). This phenomenon may be related to the fact that ischemic BRVO mostly occurs in the branches of blood vessels without involving the optic disk.

FAF, as a non-invasive imaging examination, is mainly used to detect diseases with RPE changes, such as AMD and CSC (13). We performed FAF examinations on all patients with RVO before FFA and found that FAF could clearly reveal the CME. Vujosevic et al. reported that FAF can evaluate macular edema in clinically significant cases and correlates better with OCT patterns and central field microperimetry rather than with visual acuity (17). Roesel et al. found that 59% of their patients had altered FAF in the central 500 μm, and FAF alterations correlated with the presence of cystoid spaces in the outer plexiform and inner nuclear layers on OCT (18). In another study, Vujosevic et al. reported that a greater number of intraretinal hyperreflective spots and a larger area of FAF images may indicate a more prevalent inflammatory condition in DME with a specific response to targeted treatment (19). In our study, hyperautofluorescence in the macular edema was observed in 16 of 23 patients (69.57%) with RVO in the non-ischemic group, consistent with fluorescein accumulation in the macular area on FFA. Among the remaining seven patients, six had no macular involvement. The pathology may be that the normal macula contains lutein, which can absorb light in the spontaneous fluorescence spectrum. Patients with CME show high fluorescence, as the edema cyst pushes the macular pigment and reduces its density, thereby reducing the ability to absorb stimulated luminescence. Interestingly, macular edema was observed only on FAF in 7 ischemic RVO patients. Of the remaining 19 patients, fourteen eyes had no spontaneous fluorescence due to NP areas located in the macular region, which also showed no macular edema on FFA. Neither FAF nor FFA revealed macular edema in these eyes, but it was observed on concurrent OCT. Therefore, we inferred that if macular edema in RVO patients is observed on OCT but not on FAF, the patients may be more likely to develop ischemic RVO or may already be in an ischemic phase of RVO.

In addition, OCT examination showed that the incidence of intraretinal cysts, SRF, and hyperreflective bright spots was high and similar in both the ischemic and non-ischemic groups. The number of hyperreflective bright spots in RVO patients in the ischemic group was significantly greater than that in the non-ischemic group. Hyperreflective bright spots are considered a biomarker of retinal neuroinflammatory response, primarily originating from activated microglia in the retina or aggregates of monocyte-macrophages in the blood (20). The number of hyperreflective bright spots has been shown to increase significantly under pathological conditions, and this increase may decrease when the lesion improves (21). In our study, the number of hyperreflective bright spots in patients with ischemic RVO was higher than in the non-ischemic group, consistent with previous reports of this phenomenon.

Studies have shown that DRIL is an indicator of visual prognosis after anti-VEGF treatment, and patients with DRIL usually have poor baseline and visual prognoses (22). Joltikov et al. (23) reported that diabetic subjects with DRIL have reduced retinal function compared to those without DRIL, and it may therefore be a structural biomarker for reduced retinal function in early diabetic neuroretina disease (20). Some studies have found that DRIL was associated with the non-perfusion region and the FAZ area on OCTA in DME, suggesting that its occurrence is limited to severe ischemia (24). Berry et al. concluded that DRIL on OCT was more closely associated with ischemia on FFA in acute CRVO patients and was a predictor of visual acuity through a follow-up period of more than 1 year (25). In our study, 23 patients (88.46%) presented with DRIL in the ischemic RVO group, compared with only 13 patients (56.52%) in the non-ischemic RVO group. Therefore, we believe that DRIL is associated with ischemia and can serve as an indicator of ischemic RVO.

An OCT examination can display the structure of the retina’s outer layer. The impairment of ELM and EZ integrity is believed to be related to visual prognosis in DR, RVO, and wet AMD. Studies have shown that DME patients with destruction of the ELM and EZ have poor visual prognoses after treatment (26). In our research, discontinuous ELM was found in 10 eyes (38.46%) in the ischemic group, which was higher than the corresponding rate (13.04%) in the non-ischemic group. The EZ was destroyed in 11 eyes (42.30%) in the ischemic group, which was also higher than that in the non-ischemic group (30.43%). Therefore, ELM and EZ damage can also be used as indicators of ischemia.

A p-MLM can sometimes be seen on OCT and is characterized by a dense hyperreflective line at the outer border of the inner nuclear layer. In 2013, Chu et al. found that the p-MLM sign is a useful indicator of acute ischemic retinal damage, especially in cases showing subtle or resolved retinal opacities before the onset of atrophic changes (27). Browning et al. reported that paracentral acute middle maculopathy (PAMM) and p-MLM are milder signs of ischemia than increased reflectivity of the inner retinal layers, and eyes with PAMM can evolve to exhibit the p-MLM sign (28). Furashova et al. found that p-MLM sign was present in 94% of ischemic RVO patients and 66% of non-ischemic RVO patients (29). Sebastian Bemme et al. also found that p-LML correlates with a poor prognosis for BCVA in CRVO (30). In our study, fifteen eyes (57.69%) in the ischemic group had p-MLM signs, which was higher than those in the non-ischemic group (21.73%). The p-MLM sign can also serve as an indicator of ischemia in RVO patients.

Meanwhile, we compared the CMT between two groups of RVO patients, and the results were statistically significant. CME appeared to be more severe in patients with ischemic RVO. However, in a study of ischemic BRVO, Sun et al. concluded that there was no significant difference in the severity of ME between ischemic and non-ischemic BRVOs (31). This may seem inconsistent with our findings, but their study focused on a correlation analysis between wide-angle FFA and CME. We hypothesize that wide-angle FFA may be more sensitive in detecting early ischemic RVO in cases of macular ischemia. Our research, due to a different approach, found more severe cases in patients already diagnosed with ischemic RVO compared to Sun’s study, and thus, CME was also more severe. However, SFCT did not show statistically significant differences between the RVO patient groups. Chen et al. compared choroidal blood flow in patients with ischemic RVO and found no significant difference in SFCT (32). In line with our study, we believe that RVO macular SFCT may not be sensitive enough for monitoring ischemic RVO. Greater attention should be given to the peripheral choroidal blood supply in future research. Additionally, swelling with impaired vascular exchange may reduce the visibility of SFCT changes. Therefore, it should be evaluated in combination with OCTA to assess the choroidal vascular index and other relevant parameters.

To assess indicators of ischemia, we measured the optical intensities of the inner and outer retinal layers in the three groups. We found that the optical intensity of the retina’s outer layers in the ischemia RVO group was significantly lower than in the non-ischemic and control groups. Egbert Matthé et al. recently confirmed that high-reflectivity glaucoma is indeed a characteristic of RVO ischemia (33). Qu et al. found that electrophysiological abnormalities could be more easily detected in the outer retina at an early stage in patients with ischemic RVO. These findings are consistent with our conclusions. Since OCT is more widely available than electrophysiological examinations, this suggests that outer retinal ischemia may be more readily detectable in the early stages of RVO (34). Additionally, longitudinal observations of RVO patients with recurrent CME have shown that retinal damage progresses over time (35), as evidenced by thinning of both the inner and outer retina, including the OPL (36, 37). Thus, we maintain that the reduced optical intensity of the ORL can serve as an effective indicator of ischemia in RVO patients.

Interestingly, Chen reported that the optical intensity of the inner segment ellipsoid zone/RPE was lower in the affected region in BRVO patients than in the unaffected area. In contrast, the inner retinal layers were higher in the affected region than in the unaffected region (38). The discrepancies between our findings and previous studies may be attributed to several methodological and population differences. First, earlier studies often assessed retinal reflectivity using absolute gray values without image normalization, making their measurements more susceptible to variations in signal strength, media opacity, or scan quality. In contrast, our study employed a standardized vitreous–RPE normalization approach, which may yield lower but more accurate estimates of optical intensity. Second, differences in patient characteristics, including the broader range of disease duration and higher prevalence of systemic vascular conditions in our study, may contribute to the observed variations. Finally, OCT acquisition protocols and segmentation boundaries vary across studies, further influencing layer-resolved reflectivity measurements.

This study has several significant limitations that should be considered when interpreting the results. First, the cross-sectional design prevents assessment of temporal relationships or prognostic implications, particularly regarding the evolution of imaging biomarkers such as DRIL, p-MLM, and the FAF–OCT mismatch. Longitudinal follow-up is needed to determine whether these features have predictive value for ischemic progression or treatment response. Second, CRVO and BRVO cases were analyzed together, and the disease duration varied widely, from days to years, which may influence retinal structure (39), the degree of ischemia, and FAF/OCT manifestations. Due to the limited sample size, further stratified analyses or multivariable adjustments for RVO subtype, disease duration, and systemic comorbidities were not feasible, and residual confounding cannot be excluded. Third, although we provided standardized operational definitions for key imaging biomarkers and demonstrated high inter-grader agreement, some parameters, particularly the FAF–OCT mismatch, remain exploratory. Its diagnostic value may be affected by macular hemorrhage, media opacity, or subtle FAF signal variability. Additionally, although we quantified the mismatch using contingency analysis, ROC-based assessments were underpowered in the ischemic subgroup. Fourth, multiple OCT/FAF parameters were tested without formal correction for multiple comparisons. As this study is exploratory, the reported *p*-values should be interpreted cautiously, and confirmatory studies with adjusted statistical frameworks are required. Additionally, OCTA data were unavailable for most patients (40), limiting our ability to evaluate microvascular perfusion comprehensively (33, 41, 42). Future studies with larger cohorts, uniform disease staging, and longitudinal multimodal imaging, especially OCTA, will be necessary to validate the proposed biomarkers and clarify their diagnostic utility.

## Conclusion

In summary, the presence of DRIL, the p-MLM sign, the integrity of the ELM, and the decreased optical intensity of the EZ and RPE can serve as valuable indicators of ischemia in RVO patients. CME in patients with ischemic RVO is less easily detectable on FAF compared to patients with non-ischemic RVO. This represents a new FAF finding that may help distinguish ischemic RVO when FFA is unavailable, and this lack of correspondence may provide insight into the type of RVO. These conclusions can be used to evaluate whether patients who cannot undergo FFA have ischemic changes, guiding further treatment.

## Data Availability

The raw data supporting the conclusions of this article will be made available by the authors, without undue reservation.
